# The severity of malarial anaemia in *Plasmodium chabaudi *infections of BALB/c mice is determined independently of the number of circulating parasites

**DOI:** 10.1186/1475-2875-7-68

**Published:** 2008-04-25

**Authors:** Tracey J Lamb, Jean Langhorne

**Affiliations:** 1 Division of Parasitology, National Institute for Medical Research, The Ridgeway Mill Hill, NW7 1AA, UK; 2 School of Biological Sciences, AMS Building, University of Reading, PO Box 228, Reading, RG6 6AJ, UK

## Abstract

**Background:**

Severe malarial anaemia is a major complication of malaria infection and is multi-factorial resulting from loss of circulating red blood cells (RBCs) from parasite replication, as well as immune-mediated mechanisms. An understanding of the causes of severe malarial anaemia is necessary to develop and implement new therapeutic strategies to tackle this syndrome of malaria infection.

**Methods:**

Using analysis of variance, this work investigated whether parasite-destruction of RBCs always accounts for the severity of malarial anaemia during infections of the rodent malaria model *Plasmodium chabaudi *in mice of a BALB/c background. Differences in anaemia between two different clones of *P. chabaudi *were also examined.

**Results:**

Circulating parasite numbers were not correlated with the severity of anaemia in either BALB/c mice or under more severe conditions of anaemia in BALB/c RAG2 deficient mice (lacking T and B cells). Mice infected with *P. chabaudi *clone CB suffered more severe anaemia than mice infected with clone AS, but this was not correlated with the number of parasites in the circulation. Instead, the peak percentage of parasitized RBCs was higher in CB-infected animals than in AS-infected animals, and was correlated with the severity of anaemia, suggesting that the availability of uninfected RBCs was impaired in CB-infected animals.

**Conclusion:**

This work shows that parasite numbers are a more relevant measure of parasite levels in *P. chabaudi *infection than % parasitaemia, a measure that does not take anaemia into account. The lack of correlation between parasite numbers and the drop in circulating RBCs in this experimental model of malaria support a role for the host response in the impairment or destruction of uninfected RBC in *P. chabaudi *infections, and thus development of acute anaemia in this malaria model.

## Background

Severe malarial anaemia (SMA) is a major factor contributing to malarial morbidity in humans (for review see [1,2]) and is a significant pathological feature of most rodent malaria infections [3]. During malaria infection, destruction of red blood cells (RBCs) from parasite replication is likely to contribute to the observed anaemia. However there also appears to be an interruption in the normal flow of replacement RBCs, as well as the appearance of abnormal RBCs (dyserythropoiesis) and premature destruction of uninfected RBCs (erythrophagocytosis) [4-6] that will alter the total number and relative effectiveness of RBCs in the circulation (reviewed by [7]). Although it is known that the severity of anaemia in malaria infection can be discordant with parasite replication rates, the relative contributions of RBC destruction through parasite replication and the altered replacement and persistence of functional RBCs in the circulation in SMA has not been formally assessed.

Similarly to human SMA, dyserythropoiesis and erythrophagocytosis have been observed in mouse malaria infection [8-10]. The pro-inflammatory response of the host to the malaria parasite observed in both human infections and mouse models [11-15] is thought to play a role in these processes. Tumour necrosis factor-α (TNF-α) [16-19] and macrophage migration inhibitory factor (MIF) produced by immune cells, in particular macrophages [20,21], are thought to contribute to malarial anaemia by suppressing erythroid development. Conversely, anti-inflammatory cytokines such as IL-10 may play a protective role [12,22-24]. Therefore, it is possible that under the conditions of natural malaria infections, where differences between hosts in the magnitude or skew of the immune response to malaria parasites can be observed, the contribution of the different factors determining the severity of malarial anaemia may also be altered.

The extent to which the number of parasitized RBCs (pRBCs) in the circulation correlates with the severity of malarial anaemia, or whether even in the early phase of a blood stage infection there is evidence of involvement of the host response in RBC destruction or suppression of RBC synthesis, has been examined here using blood stage infections of a mouse model of malaria, *Plasmodium chabaudi chabaudi*, which replicates in a synchronous manner approximately every 24 hours. Circulating parasite numbers have been compared with haemoglobin (Hb) levels and loss of RBC during *P. chabaudi *infections in mice. Using RAG2-/- mice lacking T and B cells, and two *P. chabaudi *clones (AS and CB) of differing virulence, the data indicate that the level of anaemia does not correlate with the number of parasite-infected RBC. Furthermore, the greater loss of RBC in the more virulent infection of *P. chabaudi *(clone CB) is not the result of a greater parasite load, but rather a decrease in the number of uninfected RBC. Therefore, an increase in the severity of anaemia in *P. chabaudi *infections of BALB/c mice appears to occur via mechanisms that are independent of parasite replication.

## Methods

### Mice

Female BALB/c and BALB/c RAG2 deficient (RAG2-/-) mice [25] aged 6 to 12 weeks were obtained from the SPF breeding facility of the National Institute for Medical Research, London, UK and maintained under conventional conditions on a 12 hr light : dark cycle. All experiments were carried out according to Home Office requirements and under approval of the Ethical Committee of the National Institute for Medical Research.

### Parasites and infection

*Plasmodium chabaudi chabaudi *clones AS and CB [26] were maintained as frozen stabilates, and parasites from the same passage were used in all experiments in this study. Mice were injected intraperitoneally with 1 × 10^5 ^pRBCs suspended in Kreb's saline and the course of parasitaemia monitored by Giemsa-stained thin blood films obtained from tail snips. The percentage of pRBCs was enumerated from counting the number of infected RBCs in 300–500 RBCs per mouse for each time point. The number of circulating parasitized RBC (pRBC or parasite numbers) were calculated by multiplying the percentage of pRBCs by the number of circulating RBCs (see below) for each mouse at each time point. For all experiments each experimental group comprised of five mice. Naive uninfected control mice were included in all experiments and were injected with Kreb's saline. Each experiment was repeated three times.

### Measurements of anaemia

The number of circulating RBCs was analysed by dilution of 5 μl tail blood in 45 μl of Krebs saline containing 100 U/ml Heparin (Leo laboratories Ltd, UK) and the samples read on a Hemavet machine (Drew Scientific) within three hours of collection. Hb was measured by diluting 5 μl of blood in 100 μl Drabkin's solution (1 g NaHCO_3_, 0.1 g K_2_CO_3_, 0.05 g KCN and 0.2 g K_3_Fe(CN)_6 _in1 litre of dH_2_O) freshly made before the start of each experiment. The amount of cyanomethoglobin formed was read at 540 nm on an ELISA reader and converted to mg/ml using a standard curve of rat Hb (Sigma). Measurements were taken on day 0 of infection, and then daily from day 3 to day 14, with a final measurement at day 19, all at the same time of day (approximately 15.00 hr) when the number of mature schizont stages was minimal.

### Statistical analysis

As measures of anaemia, the greatest drop in the number of circulating RBCs or in Hb over the course of infection was calculated by subtracting daily measurements from that measured at day 0 for each mouse. Parasitaemia was analysed as the maximum number of circulating pRBCs or percentage of pRBCs observed over the course of infection for each animal. Total parasite load was calculated from the area under the curve of circulating pRBC number for each animal using Prism software (Prism Inc). In each of the three experiments carried out with the two different clones of *P. chabaudi*, some animals infected with clone CB died (collective values over the three experiments are days 10, 12 and 15 PI: 1 animal per day; days 9 and 13 PI: 2 animals per day; day 11: 4 animals). Only one animal infected with clone AS died at day 13 in experiment 1. For all these animals the maximum values of parasitaemia and anaemia recorded before death analysed.

Analysis of variance (ANOVA) (Minitab Inc.) [27] was used to meta-analyse the combined data from three different experiments. Experiment and a term to check for any experimental interaction between the mouse strain or parasite clone were included as predictor factors to account for between-experimental differences and verify that all experiments reached the same conclusion. The naive animals were removed from all the statistical analyses since they did not suffer any discernible anaemia. Analysis of the residuals from the maximal model of the ANOVA containing all first order interactions confirmed that the transformed data accorded with the assumptions of parametric tests. The peak pRBC number for each mouse was logarithmically transformed prior to analysis in order to satisfy these requirements of parametric testing. In all cases P values < 0.05 were considered statistically significant and unless otherwise stated significant P-values are from the minimal model; non-significant P-values are from the models from which they were removed.

## Results

### The number of circulating pRBCs does not correlate with the severity of anaemia in either BALB/c or RAG2-/- animals over the first 9 days of infection

To determine whether parasite-mediated destruction of RBC was responsible for the acute anaemia observed in *P. chabaudi *AS infections, parasitaemia and anaemia were compared in infected RAG2-/-, lacking T and B cells, and immunocompetent BALB/c mice infected with *P. chabaudi *clone AS. Since RAG2-/- are unable to control the growth of malaria parasites and generally die of a fulminating parasitaemia between 10 to 12 days post-infection [28] measurements were taken only up to day 9 of infection, prior to the peak of infection and before anaemia was maximal.

**Figure 1:**
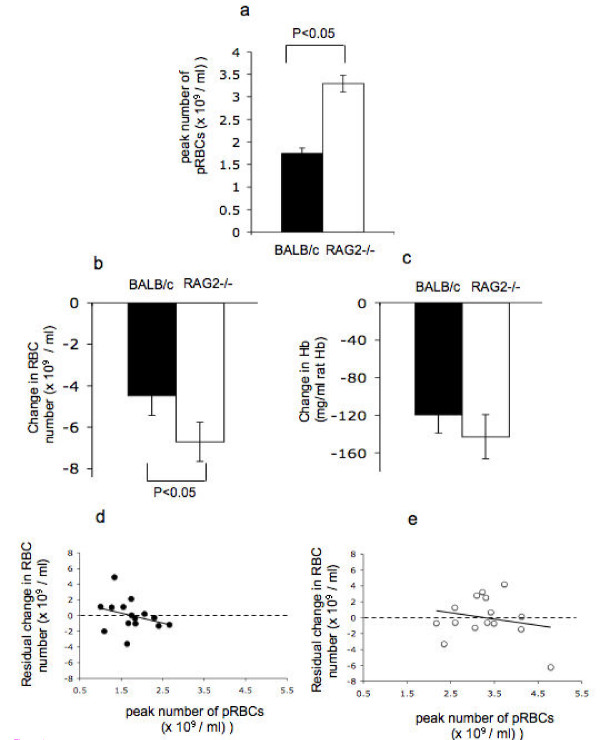
**Comparison of anaemia and peak parasitaemia in *P. chabaudi *(AS) infected recombinase activating gene 2 -/- (RAG2-/-) and BALB/c mice**. a) Peak number of circulating pRBCs measured over the first 9 days of infection; b) Drop in the number of circulating RBCs and c) Drop in Hb level of the blood relative to day 0 measurements in BALB/c and BALB/c RAG2-/- animals. The bars in a) to c) represent the average, and the error bars the SEM, of the cumulative values of 15 BALB/c AS-infected animals (black square) 15 RAG2-/- AS- infected animals (open square) from 3 experiments. d) and e) Residual amount of anaemia (once experiment has been taken into account) in *P. chabaudi *infection of BALB/c mice (black circle) and RAG2-/- mice plotted (open circle) against the peak number of circulating pRBCs for each mouse. In d) and e) the solid line represents the best-fit linear regression line. The dashed line highlights the residual anaemia observed once the data is normalized for experimental variation in the magnitude of RBC loss (ANOVA model "RBC loss = experiment"). Therefore the solid best-fit regression line shows the extent to which peak pRBC numbers correlate with the drop in circulating RBCs once experiment has been accounted for (residual change in RBC number). The black dots represent BALB/c animals and the white dots represent RAG2-/- animals.

As expected the peak number of pRBCs was consistently higher in RAG2-/- animals compared with BALB/c control mice (Figure 1a, mouse strain: F_1,28 _= 50.53 P = 0.000) suggesting that the presence of an acquired immune system contributed to control of parasite growth even at this early stage. During this period, the drop in the number of RBCs, but not the drop in Hb levels, was significantly greater in RAG2-/- mice compared with BALB/c mice (Figures 1b and 1c, RBC loss: F_1,26 _= 6.14 P = 0.020, Hb drop: F_1,26 _= 2.82 P = 0.105). Despite the greater loss of RBC in RAG2-/- mice there was no significant correlation between peak numbers of pRBC and the drop in circulating RBC for either RAG2-/- or BALB/c mice (Figures 1d and 1e, F_1,11 _= 0.55 P = 0.475 and F_1,11 _= 1.79 P = 0.208 respectively) suggesting that parasite destruction alone was not responsible for the reduction in RBC numbers.

### *Plasmodium chabaudi *clone CB induces more anaemia than clone AS, despite a similar parasitaemia curve

It has been shown previously that the severity of anaemia in mice infected with *P. chabaudi *varies with the clone of *P. chabaudi *parasite used [29]. It was, therefore, of interest to determine whether anaemia caused by different *P. chabaudi *clones could be ascribed to an increase in direct RBC destruction by the more virulent clone, CB. The loss of RBC and numbers of circulating pRBC in *P. chabaudi *AS and CB infections of BALB/c mice were compared.

**Figure 2:**
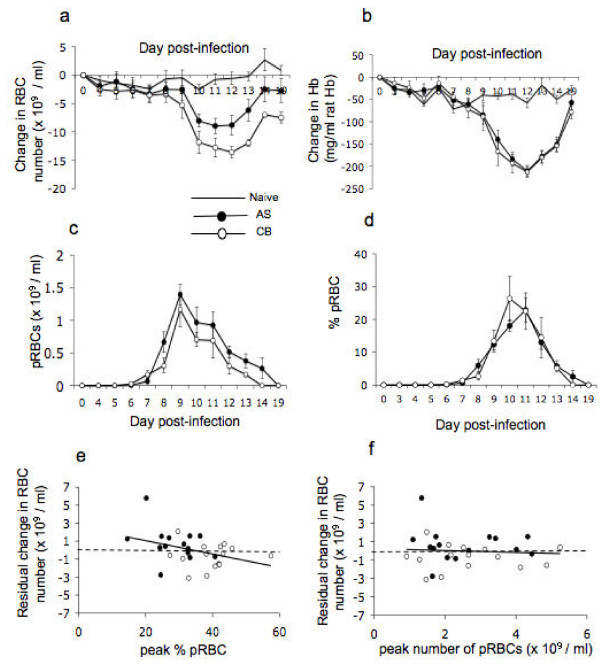
***P. chabaudi *clone CB induces more anaemia than clone AS in BALB/c mice**. a) Drop in the number of circulating RBCs; b) drop in the Hb level in the blood over the course of the primary infection; c) Number of circulating parasites and d) % of pRBC over the same time scale in AS-infected mice (black circle), CB-infected mice (open circle) and naive control animals (-). For each day the circles in a) to d) represent the average and the error bars the standard error of the mean (SEM) of 5 animals from experiment 2. e) and f) residual number of circulating RBCs after experiment and clone have been taken into consideration plotted against peak percentage parasitemia (e) or peak number of pRBC (f) for all three experiments. Each symbol represents the values for a single mouse infected with clone AS (black circle) or clone CB (open circle). The solid lines represent the best fit linear regression line in each case, and the dashed lines highlight the residual anaemia observed once the data is normalized for experimental and clonal variation in the magnitude of RBC loss (ANOVA model "RBC loss = experiment+clone").

Maximum loss of RBC occurred at a similar time in both infections (Table 1; combined data over three experiments: F_1,26 _= 3.48 P = 0.074), but was significantly greater in mice infected with clone CB than clone AS (Figure 2a and Table 1; combined data: F_1,26 _= 4.786 P = 0.037). Unlike the loss of RBC, the maximum drop in Hb was not consistently different between infections with *P. chabaudi *AS or CB (Figure 2b and Table 1; interaction term between clone and experiment: F _2,24 _= 8.03 P = 0.002). The drop in Hb concentration and drop in circulating RBC numbers were not correlated with each other (F_1,26 _= 2.08 P = 0.161; data not shown) indicating that the amount of Hb during malaria infection may not be the same in every RBC when comparing infection with AS and CB.

**Table 1:** Parasitaemia and anaemia data in primary *P. chabaudi *AS or CB infections of BALB/c mice.

CLONE	Experiment 1		Experiment 2		Experiment 3	
	AS	CB	AS	CB	AS	CB
	AS	CB	AS	CB	AS	CB
Peak Parasite Number (×10^9 ^/ml) ^(1)^	3.9 ± 0.2	4.0 ± 0.5	1.5 ± 0.1	1.4 ± 0.2	2.1 ± 0.2	2.6 ± 0.3
Day of Peak Parasite Number ^(2)^	10.8 ± 0.2	9.2 ± 0.2	9.0 ± 0.3	9.4 ± 0.4	7.0 ± 0.5	7.4 ± 0.4
Parasite Load ((pRBC × 10^9^/ml)days) ^(1)^	13.1 ± 1.3	16.4 ± 2.8	5.1 ± 0.6	3.1 ± 0.3	7.1 ± 0.5	6.0 ± 0.4
Peak % of pRBC ^(1,3)^	30.1 ± 1.8	42.6 ± 0.9	23.3 ± 2.8	31.8 ± 1.9	33.4 ± 2.7	43.9 ± 3.6
Day of peak % of pRBC ^(1,3)^	10.8 ± 0.2	9.2 ± 0.2	10.8 ± 0.2	10.6 ± 0.4	8.4 ± 0.4	8.2 ± 0.4
Maximum RBC loss (×10^9 ^/ml) ^(1,3)^	7.4 ± 0.4	9.1 ± 0.5	11.5 ± 1.4	13.6 ± 0.9	9.2 ± 0.4	9.4 ± 0.3
Day of greatest loss in RBC ^(1)^	11.6 ± 0.2	10.2 ± 0.4	11.2 ± 0.4	11.6 ± 0.5	9.6 ± 0.2	8.8 ± 0.4
Maximum Hg drop (mg/ml) ^(2)^	54.8 ± 12.6	152.8 ± 22.8	210.4 ± 11.0	219.0 ± 9.8	230.4 ± 6.5	242.1 ± 4.7
Day of greatest Hg drop ^(1,3)^	12.0 ± 0.8	10.4 ± 0.2	12.0 ± 0	11.6 ± 0.5	9.8 ± 0.4	9.0 ± 0.4

The values represent the averages ± SEM from 5 animals

1) Significant difference in magnitude between experiments (P < 0.05)

2) Significant difference in clonal behaviour between experiments (P < 0.05)

3) Significant difference between clones (P < 0.05)

Despite the greater RBC loss in *P. chabaudi *CB infections, the peak numbers of circulating pRBCs were not significantly different in the two infections (Figure 2c and Table 1; combined data: F_1,26 _= 0.63 P = 0.435). The peak number of circulating pRBCs occurred at similar times for both clones although this was not uniform between the three experiments (Table 1; interaction term between experiment and clone F_2,24 _= 5.00 P = 0.015). Total parasite load (as calculated by the area under the parasite curve) similarly was not significantly different between the clones (Table 1; combined data: F_1,26 _= 0.47 P = 0.497). Together these data indicate that clone CB induces more anaemia than clone AS over the course of infection in BALB/c mice, despite similar numbers of pRBC and a similar parasite load.

### Peak percentage of pRBCs, but not peak number of pRBC, correlates with the drop in circulating RBCs in *P. chabaudi *infection

Although the number of circulating pRBCs in CB and AS infections was not significantly different (Figure 2c and Table 1), the maximum percentage of circulating pRBCs in mice infected with clone CB was observed earlier and was significantly higher than in AS-infected mice (Figure 2d and Table 1; Day of maximum parasitaemia combined data: F_1,26 _= 0.63 P = 0.043, maximum percentage of pRBCs combined data: F_1,26 _= 29.33 P = 0.000) indicating that there were fewer RBC available for infection in CB infections. In line with these data, the maximum percentage parasitaemia over the course of infection was strongly correlated with anaemia (Figure 2e, peak percentage of pRBCs F_1,26 _= 6.72 P = 0.015), whereas there was no correlation between loss of RBC and peak number of pRBC across both clones (Fig 2f, F_1,24 _= 0.00 P = 0.993). Together these data suggest that parasite destruction of RBCs is not a major factor leading to variation in anaemia within CB and AS infections, and that lack of availability of RBCs leading to higher percentage of pRBCs in CB infections may explain the greater anaemia in this infection.

In contrast to the correlation of the peak percentage of pRBCs with the drop in circulating RBCs, the peak number of pRBCs (but not the peak percentage of pRBCs) did correlate with drop in haemoglobin in the blood (peak pRBC number combined data: F_1,23 _= 12.02 P = 0.000; peak percentage of pRBCs combined data: F_1,22 _= 0.43 P = 0.521). These results provide further evidence that the two measures of anaemia (loss of RBC and Hb levels) do not behave in the same manner, and again suggest that RBCs in the circulation in malaria-infected mice can contain different levels of Hb.

## Discussion

The data from the experiments in this study indicate that the acute severe anaemia accompanying a blood stage infection with *P. chabaudi *in mice is not primarily the result of direct parasite destruction of infected RBCs. In particular, during infections with two clones of *P. chabaudi *that differ in virulence, the drop in circulating RBC numbers was clearly not associated with the total number of circulating parasites, but rather with the percentage of infected RBCs suggesting that it is availability of new RBC that is limiting in this infection model. As this association also occurred in RAG2-/-immunodeficient mice, the mechanism governing availability of RBCs do not seem to require an adaptive immune response.

The reduced number of uninfected RBCs in acute *P. chabaudi *infections could be the result of the host response to the infection, which leads to impaired replacement of RBC (erythropoiesis), the premature destruction of uninfected RBCs (erythrophagocytosis) or results in the production of defective RBCs (dyserythropoiesis) which would be removed by the reticular endothelial system [7-10,30]. In *P. chabaudi *(AS) infections, impaired erythropoiesis has been shown to be due, in part, to the reduced ability of RBC precursors to respond to erythropoietin (EPO), resulting in limited replacement of RBCs and thus exacerbation of anaemia observed during infection [31]. An increase in the removal of either infected RBC or erythrophagocytosis via opsonizing immunoglobulin cannot wholly explain RBC loss described in this study, as a similar loss of RBC also occurs in *P. chabaudi *RAG2-/- mice, in the absence of an acquired immune system. However, it is still possible that complement components activated via the alternate pathway could facilitate erythrophagocytosis by allowing the recognition of erythrocytes via macrophage complement receptors [32]. Such a mechanism may normally facilitate steady state haemostasis, but may be enhanced under the immune conditions of a malaria infection.

Infection with the more virulent CB clone of *P. chabaudi *resulted in a greater reduction in RBC number than the less virulent clone AS, despite comparable numbers of parasites (Figure 2c) and invasion rates (data not shown). Across the AS and CB-infected BALB/c mice, pRBC numbers peaked between 1.4 -4 × 10^9 ^pRBCs with a concomitant loss of 7.4 – 13.6 × 10^9 ^RBCs in circulation (Table 1). Therefore, anaemia in *P. chabaudi *infections cannot be entirely due to haemolysis of RBCs due to parasite replication. Since *P. chabaudi *can sequester in the liver [33], it is possible that the circulatory pRBCs are not entirely representative of parasite population numbers. However, this was controlled for by measuring numbers of pRBCs at 3 pm in the normal light cycle, a time when the sequestering schizont stage of *P. chabaudi *is not present in this synchronous malarial infection [34,35]. Alternatively, it is possible that selective invasion of *P. chabaudi *for young normocytes and reticulocytes may erode the replenishment of circulating RBCs. However current evidence suggests that *P. chabaudi *only invades younger cells when more mature normocytes are in short supply [36]. Additionally, if this explained the clonal differences in the severity of anaemia, we should concomitantly see differences in parasite load between the clones, with an extended parasitaemia in one of the clones when normocytes are in short supply. It seems more likely that it is the differential induction of the host response by the CB clone that is responsible for the greater degree of anaemia observed in this infection.

Rodent malaria parasites can activate mouse macrophages [37,38] to produce the cytokines IL-12 [37], TNF-α and MIF [20] probably via recognition of parasite ligands by pattern-recognition receptors (PRRs) [39]. These cytokines have all been implicated in the immunopathogenesis of anaemia in mouse malaria infections [3]. It is thus possible that the response of innate cells such as macrophages, dendritic cells or NK cells could contribute to the host response and therefore to anaemia. It would therefore be of great interest to determine whether impaired erythropoiesis or erythrophagocytosis in CB-infected mice is greater than that measured in AS-infected animals. Since the peak percentage of parasitaemia occurred earlier in CB than in AS infections it might be that an impaired response to EPO by erythroid progenitor cells is detectable at an earlier time point in CB-infected animals. A detailed examination of the nature of the host response to the different *P. chabaudi *clones would clarify whether cells of the innate system play a role in the development of anaemia.

The discrepancy between the total number of circulating parasites and the percentage parasitaemia in the clonal infections described here highlights the need for caution in the interpretation of percentage of infected RBC as a measure of parasitaemia. Percentage parasitaemia is confounded by the level of anaemia and thus can be misleading as a determinant of parasite numbers. It appears that the cause of the differences in anaemia between the two clones is due to differences in the number of uninfected RBCs in circulation in CB-infected and AS-infected mice.

An interesting finding of this study was that a reduction in the number of circulating RBC did not necessarily correlate with a drop in the levels of Hb in the blood. Infected RAG2-/- mice had lower circulating numbers of RBCs than infected BALB/c, yet displayed similar levels of Hb. Furthermore, there was no correlation between the drop in RBC numbers and Hb in mice infected with either of the two *P. chabaudi *clones. This finding may be related to different levels of circulating reticulocytes between RAG2/- and BALB/c animals, and between BALB/c animals infected with clone AS or CB. Reticulocytes are elevated in *P. chabaudi *AS infection as a response to the drop in circulating RBC number [36]. Although we did not measure circulating reticulocyte numbers in the infections in this study, we would predict that both RAG2-/- AS-infected animals, and BALB/c CB infected mice would have a greater impairment of haematopoiesis and thus a lesser number of reticulocytes in the circulation than AS-infected BALB/c mice. If reticulocytes have reduced haemoglobin levels until they fully mature into RBCs, then it follows that the AS-infected animals will have lower circulating Hb levels relative to the number of circulating RBCs compared with AS-infected RAG2-/- or CB-infected BALB/c mice. Regardless, these results emphasize that any investigation of malarial anaemia should include more than one measurement of anaemia.

## Conclusion

In conclusion, we have found in this study that in the *P. chabaudi *model of malarial anaemia, circulating numbers of pRBCs do not always account for the severity of anaemia observed. Other mechanisms affecting adequate turnover and maintenance of RBC numbers in the circulation appear to play a greater role in determining the severity of anaemia in *P. chabaudi *infection than parasite destruction of RBCs through replication. To understand the basis of SMA, future research into the mechanisms governing RBC generation in malaria infection is merited.

## Abbreviations

RBC, red blood cell; pRBC, parasitized red blood cell; RAG2-/-, recombination activating gene 2 deficient.

## Competing interests

The authors declare that they have no competing interests

## Authors' contributions

The data was collected and analysed by TL. Both authors contributed to the experimental design, interpretation of the data and writing of the manuscript. Both authors read and approved the final manuscript.
